# Reviewed article: Freitas TCR, Soares AB, Figueiredo BNS. Evaluation of prophylaxis protocols and spatial distribution of humans wounded by bats: a descriptive study, Palmas, 2013-2023. Epidemiol Serv Saude. 2026:35;e20250557

**DOI:** 10.1590/S2237-96222026v35e20250557.b

**Published:** 2026-03-16

**Authors:** Vanessa Santos de Arruda Barbosa

**Affiliations:** 1 Universidade Federal de Campina Grande, Cuité, PB, Brazil

## Peer review B

## Questionnaire

Does the manuscript contain new and significant information to justify publication? Yes

Does the Abstract (Summary) clearly and accurately describe the content of the article? Yes

Is the problem significant and concisely stated? Yes

Are the methods described comprehensively? Yes

Are the interpretations and conclusions justified by the results? Yes

Is adequate reference made to other work in the field? Yes

## Manuscript Structure

Length of article is: Adequate

Number of tables is: Adequate

Number of figures is: Adequate

Please state any conflict(s) of interest that you have in relation to the review of this paper. None

Interest: Good

Quality: Good

Originality: Good

Overall: Good

Recommendation: Minor Revision

## Comments

O manuscrito é original e de importância para o avanço da temática, trazendo contribuições na área da saúde coletiva. No entanto, sugiro observação dos seguintes pontos abaixo:

Introdução: No 50 parágrafo da introdução é relatado que “Entre 2010 e setembro de 2024, 48 casos de raiva humana foram notificados no Brasil, sendo 24 (50%) destes causados por morcegos”. No entanto, na referência citada do Ministério da Saúde, os casos por morcego foram 22, no intervalo supracitado.

Metodologia: No último parágrafo, inserir como referência, o número da nota técnica no qual se baseia o protocolo de profilaxia antirrábica vigente.

Em todo o texto:

Substituir o termo “agressão” ou “agressões” por morcegos no título e ao longo do texto para os termos: “acidentes” ou “agravos” ou “transmissão” por morcegos. Embora o Ministério da Saúde utilize a nomenclatura “agressão” e “animal agressor”, essa é uma visão antropocêntrica e equivocada sobre o comportamento dos quirópteros que se encontram, muitas vezes, em situações desfavoráveis de perda de habitat e com poucas presas naturais. Ressalta-se ainda que pressões antrópicas levam a comportamentos de estresse e doença, os quais podem gerar o contato com os humanos. Dessa forma, o ajuste do termo é fundamental para dar uma visão mais ecológica e alinhada com os preceitos de Saúde Única.

